# The *Tomato yellow leaf curl virus* V2 protein forms aggregates depending on the cytoskeleton integrity and binds viral genomic DNA

**DOI:** 10.1038/srep09967

**Published:** 2015-05-05

**Authors:** Adi Moshe, Eduard Belausov, Annette Niehl, Manfred Heinlein, Henryk Czosnek, Rena Gorovits

**Affiliations:** 1 Institute of Plant Sciences and Genetics in Agriculture and the Otto Warburg Minerva Center for Agricultural Biotechnology, The Robert H. Smith Faculty of Agriculture, Food and Environment, The Hebrew University of Jerusalem, Rehovot, Israel; 2 Institute of Plant Sciences, Volcani Center, Bet Dagan, Israel; 3 Institut de Biologie Moléculaire des Plantes du CNRS, Université de Strasbourg, Strasbourg, France

## Abstract

The spread of *Tomato yellow leaf curl virus* (TYLCV) was accompanied by the formation of coat protein (CP) aggregates of increasing size in the cytoplasm and nucleus of infected tomato (*Solanum lycopersicum*) cells. In order to better understand the TYLCV-host interaction, we investigated the properties and the subcellular accumulation pattern of the non-structural viral protein V2. CP and V2 are the only sense-oriented genes on the virus circular single-stranded DNA genome. Similar to CP, V2 localized to cytoplasmic aggregates of increasing size and as infection progressed was also found in nuclei, where it co-localized with CP. V2 was associated with viral genomic DNA molecules, suggesting that V2 functions as a DNA shuttling protein. The formation and the 26S proteasome-mediated degradation of V2 aggregates were dependent on the integrity of the actin and microtubule cytoskeleton. We propose that the cytoskeleton-dependent formation and growth of V2 aggregates play an important role during TYLCV infection, and that microtubules and actin filaments are important for the delivery of V2 to the 26S proteasome.

Many plant and animal viruses induce the formation of insoluble aggregates/inclusion bodies inside infected cells. These structures usually contain viral and host proteins, and can vary in location, size, content and biological function^1^,^2^. Many plant and animal viral proteins interact with, and move along the cytoskeleton and endoplasmic reticulum (ER) membranes, as monomers, dimers and protein complexes^1^,^3^,^4^. The formation of virus-induced structures inside the infected cell, such as replication complexes, viral factories (VFs), and inclusion bodies, often depends on trafficking of host and viral proteins along the cytoskeleton. These structures can recruit host components associated with defenses against infection and cell stress. For example, components of protein quality control such as chaperones and the 26S proteasome were shown to be localized in VFs^1^,^5^.

The interaction with cytoskeleton elements was studied mostly, but not only, with RNA viruses. The involvement of cytoskeletal elements in virus inter- and intra-cellular movement and/or in the formation of VFs was shown for many plant viruses^3^. Associations with actin filaments may occur in the context of interactions with the ER for directed and efficient motor-mediated transport of membrane-associated proteins^1^,^6^. For example, *Potato virus X* (PVX) TGBp1, TGBp2, TGBp3, and coat protein are required for virus cell-to-cell movement^7^,^8^,^9^. TGBp2 forms granular ER-derived vesicles associated with actin filaments^7^. TGBp3 is recruited to these vesicles^8^ and these complexes are later found in a large perinuclear aggregate named X-body, which serves as a viral replication site similar to animal VFs^9^. TGBp1 is a major player in the remodeling of actin needed for the formation of X-bodies^9^.

Actin filaments and associated myosin motor proteins are also important for *Tobacco mosaic virus* (TMV) and facilitate the transport of the viral MP and MP-containing viral replication complexes (VRCs) along the ER to the plasmodesmata (PD)^3^,^4^,^6^. In addition, the MP of this virus interacts with microtubules that act as a dynamic scaffold for the development of the VRCs and their guidance along the ER/actin network^10^. The VRCs form particularly at sites at which microtubules intersect with the ER-actin network. At later stages, these microtubule–anchored VRCs form large aggresome-like structures that may act as VF, and MP is released from these structures for degradation by the 26S proteasome^11^. Dependent on MP expression level, the extraction of MP may result in its accumulation along microtubules^11^, which may occur when the ubiquitin-proteasome system is saturated^13^. The association of microtubules and MP with both the formation and subsequent degradation of VFs suggests that TMV uses MP to exploit aggresomal processes for recruitment and subsequent disposal of viral and host factors and membranes^10^,^12^.

The movement protein of *Potato leafroll virus* (PLRV) MP17 localizes to PD of infected source cells in an actin filaments/ER-Golgi-dependent manner, while in sink tissues, it is degraded in a microtubule/proteasome-dependent manner. Inhibition of the proteasome in sink leaves leads to the formation of a single large aggregate per cell, resembling the mammalian aggresome^14^.

Microtubules play an important role also in the formation of electron lucent inclusion bodies (ELIBs) from electron dense inclusion bodies (EDIBs) during infection with the double stranded (ds) DNA virus *Cauliflower mosaic virus* (CaMV)^15^,^16^. Aphid feeding on an infected leaf triggers a rapid and massive influx of tubulin into the transmission body, which then burst open, dispersing virus particle throughout the cell, where they become more accessible to the insect^17^. Consequently, it appears that interaction of viral proteins with microtubules is a general feature of plant viruses, and is important for the development of infection as well as for efficient virus uptake by insect vectors^10^. We have recently described the progressive aggregation of *Tomato yellow leaf curl virus* (TYLCV) coat protein (CP) in tomato (*Solanum lycopersicum*) leaf cells^18^. TYLCV is a single stranded (ssDNA) plant virus (~2,800 nucleotides) (genus *Begomovirus*, family *Geminiviridae*) transmitted in nature by the whitefly *Bemisia tabaci*^19^. At the early stages of whitefly-mediated infection, CP appeared in the cytoplasm of phloem-associated cells in the form of small aggregates. With the progress of infection, these aggregates increased in size and formed large aggregates in the cytoplasm and later in the nucleus. Nuclear aggregates contained viral genomic ssDNA, viral dsDNA formed during replication, viral CP-DNA complexes and infectious particles, suggesting that viral replication and assembly take place in large nuclear aggregates, which may be the plant equivalent of animal VFs^18^. TYLCV-induced CP aggregation processes were correlated with the development of the viral disease.

In the current study, we investigated the subcellular accumulation pattern and properties of V2, a protein encoded by the same ssDNA genome as CP and transcribed from the same promoter as CP^20^. Previous studies using *Nicotiana tabacum* protoplasts showed V2 to be localized mainly in the cytoplasm, around the nucleus and at the cell periphery, as well as in punctate bodies distributed throughout the cytoplasm^21^. This viral protein is required for systemic infection and possibly plays a role in the CP-mediated transport of viral DNA into the nucleus^21^,^22^. V2 also acts as a RNA-silencing suppressor, counteracting TYLCV-induced plant RNA silencing by impairing SGS3 function in the silencing pathway^23^,^24^. In addition, V2 was shown to recognize and directly bind the tomato CYP1 protein, a member of the papain-like cysteine protease family, which is involved in plant defense against diverse pathogens^25^.

Here, we show that TYLCV V2 forms aggregates in tomato tissues and is able to bind TYLCV genomic ssDNA (but not dsDNA replication form). We also demonstrate that V2 associates with the cytoskeleton; furthermore intact actin filaments and microtubules are needed to deliver V2 to the 26S proteasome for degradation. We propose that the cytoskeleton is required for the development of V2 aggregates, a parameter of the successful TYLCV invasion of plant cells. Moreover, our data indicate that V2 aggregates are targeted for degradation by the 26S proteasome.

## Materials and Methods

### Sources of virus, insects and plants

TYLCV from Israel (GenBank X15656.1) was maintained in tomato plants (cv. Daniella) by whitefly-mediated inoculation^20^ and served as source of CP and V2. Whiteflies (*B. tabaci* B biotype) were reared on cotton plants grown in insect-proof cages at 26 °C, as described^26^. Tomato plants at their 3-5 true leaf stage were caged with viruliferous whiteflies (about 50 insects per plant at the onset of infection) for the duration of the experiments. Whiteflies were discarded before tissue sampling*. Nicotiana benthamiana* plants, four weeks after sowing, were used for infiltration experiments. All plants were grown in a greenhouse kept at 24 °C (10 h, light) to 18 °C (14 h, dark).

### Plasmids

The PCR-amplified V2 ORF (nucleotides 314-664) was cloned into the *NdeI* and *BamHI* sites of pET-14B (Novagen). The recombinant plasmid pET-V2 was used to transform *E. coli* cells, strain BL21 (DE3). pBIN19-V2:GFP was a gift from Prof. Y. Gafni, (Volcani Center, Israel)^23^. The microtubule marker MAP4:RFP has been described earlier^27^.

### *In vitro* immuno-detection of viral and plant proteins

Western blotting was performed according to standard procedures. The source of antibodies was as follows: anti-TYLCV-V2 primary antibody was prepared similarly to the anti-TYLCV-CP^18^; anti-Histone H3, anti-HSP70 cytoplasmic, and goat peroxidase coupled secondary antibodies were purchased from Agrisera (Sweden); anti-OE33, a 33-kDa subunit of photosystem II oxygen evolving complex, was a gift from Prof. Z. Adam (The Hebrew University of Jerusalem, Israel). Incubation with antibodies was followed by ECL detection (Amersham, UK).

### Extraction and fractionation of proteins from tomato leaves and stems

Leaves (pooled from three plants) were collected and drill-homogenized in detergent-free buffer H (50 mM Tris-HCl pH 7.5, 80 mM KCl, 10 mM MgCl_2_, 0.2 mM EDTA, 1 mM dithiothreitol and Complete Protease Inhibitor Mixture - Roche, Mannheim, Germany). Stem pellet after spin down was re-suspended in buffer H and drill-homogenized. The leaf and stem homogenates were filtered through a cellulose membrane (N 334151, Schleicher and Schuell, Dassel, Germany) to separate debris and insoluble cell wall fraction from the filtrate. Proteins in cell debris and cell wall fraction were not gradient-separated further; the whole fraction was named Pellet (P). The filtrate was subjected to centrifugation at 3000 *g* for 20 min to obtain a pellet (P3) and a supernatant (S3). Native total proteins were isolated as follows: leaves and stem spin down pellet (pooled from three plants) were drill-homogenized in buffer H supplemented with 0.5% Nonidet P40. Homogenates were incubated on ice for 45 min, vortexed and centrifuged at 1200 *g* for 10 min at 4 °C. The supernatant containing native proteins was further analyzed^5^. Cytosolic and nuclear protein fractions were prepared as described before^18^. For sucrose density gradient, extracts of native proteins (0.5 ml) were layered on 10 ml linear 10–50% sucrose gradients^28^. After 20 h centrifugation at 104,000 g at 4 °C (Beckman SW27 rotor), the gradients were fractionated into 10 aliquots. For protein immunodetection, aliquots from each gradient were precipitated by ice-cold 10% TCA, washed with cold acetone and dried. Pellets were dissolved in SDS-PAGE buffer, boiled for 10 min and Western blot analyzed. Each PAGE sample contained at least 100 μg of total tomato proteins. Each experiment was repeated at least five times independently.

### Visualization of TYLCV CP and V2 in tomato leaves and stems

For histological analyses, cross sections of tomato leaves and stems (cut into 0.5 cm squares) were processed as described^18^. Briefly, after fixation in 4% paraformaldehyde in MTSB (50 mM PIPES, 5 mM EGTA, 5 mM MgSO_4_, pH 7) leaf and stem samples were embedded in wax (PEG 400 distearate and 1-hexadecanol – both from Sigma – mixed at a ratio of 9:1). Fifteen micrometer-thick wax-embedded tissues were sectioned with a microtome (HM340E, Waldorf, Germany), rehydrated and blocked for 1 h at 25 **°**C in 2% BSA/MTSB prior to incubation for 18 h at 4 **°**C with anti-TYLCV-V2 primary antibody diluted 1:100 and/ or with anti-TYLCV-CP diluted 1:100 in 2% BSA/MTSB. After washing with MTSB the samples were incubated for 1.5 h at 25 **°**C with a Cy3- and Cy2-conjugated anti-rabbit secondary antibody (Jackson Immunoresearch, USA) diluted 1:200. The samples were inspected using a stereoscopic fluorescence zoom microscope (SMZ1500, Nikon, Japan) and fluorescence microscope (Eclipse 80i, Nikon, Japan); V2 was detected as red fluorescent signal, CP was detected as green fluorescent signal. Plant nuclei were stained with DAPI (Thermo Scientific DAPI, Pierce Protein Research Product), at 1 μg/ml for 20 min at 25 **°**C, and detected as blue fluorescent signal.

### Transient expression of fusion proteins in plants

*N. benthamiana* leaves were infiltrated with *Agrobacterium tumefaciens* transformed with the expression plasmids. The bacteria were grown for 48 h at 28 °C in LB medium containing antibiotics. The culture was centrifuged at 4,000 × *g* for 8 min, and the pellet was re-suspended in water to a final optical density (OD 600) of 0.1-0.6. The suspension was used to infiltrate the underside of young leaves of 5-weeks-old plants using a syringe. The GFP/RFP-fusion proteins were visible under the fluorescence microscope 48 h after infiltration. For confocal imaging, we used an Olympus IX 81 inverted laser scanning confocal microscope (Fluoview 500) equipped with an argon ion laser and a 60 × 1.0 N.A. PlanApo water-immersion objective. Excitation/emission wavelengths were 488 nm/505 to 545 nm for GFP and 543 nm/585 to 615 nm for RFP. Plant nuclei were stained with DAPI infiltrated into leaves 30 min prior to detection at excitation/emission wavelengths of 350 to 470.

### Measurement of number and size of aggregates

To determinate the number and cross-sectional areas of aggregates, images were imported into ImageJ ( http://imagej.en.softonic.com/). The number of aggregates per image-area and their size were measured using the “threshold” and “particle analyzer” functions of the program.

### Inhibitor treatments

Oryzalin, latrunculin B (latB) (Sigma, USA) and MG132 (Calbiochem, Germany) were dissolved in dimethyl sulfoxide (DMSO) to prepare stock solutions of 100 mM. For *in vivo* treatment of *N. benthamiana* leaves, stock solutions were diluted in water to prepare solutions of 20 μM oryzalin, 10 μM latB and 50 μM MG132. The freshly prepared solutions were syringe infiltrated into the abaxial leaf side 24 h after agroinfiltration of V2:GFP. Leaves were examined under the microscope 15-20 h after inhibitor infiltration. Control infiltrations were performed using the same final concentration of DMSO without the drug. For tomato *in planta* treatment, detached leaflets from infected tomato plants (49 dpi) were placed in micro-tubes containing 20 μM oryzalin, 10 μM latB and/ or 50 μM MG132 (one leaflet per tube, the tip of the petiole soaking in the solution). Water with the same final concentration of DMSO but without the drugs was used as control. The solutions were replaced daily for 4 days. Proteins were extracted, separated on sucrose gradients and immuno-detected as described.

### Detection of TYLCV DNA-V2 complexes

Interaction between V2 and total DNA extracted from leaves of TYLCV-infected tomato plants was detected by immuno-capture-PCR (IC-PCR)^29^. Briefly, PCR tubes were coated with anti-V2 antiserum (diluted 1:500) and incubated during 1 h at room temperature with purified V2 (100 μg) and DNA extracted from infected plants (10 μg). If required, DNA extracts were treated with S1 nuclease (3 units/sample for 5 min at room temperature) prior to incubation with V2. PCR amplification of the viral DNA bound to V2 (itself bound to the V2 antibody-coated tubes) was performed with TYLCV-specific CP primers as described^18^. The experiments were repeated 3 to 5 times. Alternatively, TYLCV DNA-V2 complexes were detected by immuno-precipitation followed by Southern blot analysis. Protein A-Sepharose beads (Pharmacia, Sweden) were incubated with an excess of anti-V2 serum, followed by washing to remove unbound antibodies. Homogenates from infected tomato leaves were added to Protein A-bound anti-V2 antibody and incubated for 10 h at 4 °C. The resulting eluates were treated with phenol/chloroform to extract DNA. If required, extracts were thereafter treated with S1-nuclease. The extracted DNA was subjected to 1% agarose gel electrophoresis, blotted onto Hybond-XL membranes (Amersham Biosciences, UK) and hybridized with a TYLCV CP amplicon labeled with digoxigenin (Roche Diagnostics, Indianapolis, IN, USA). Detection of the viral DNA was performed using the CDP-Star kit (Roche Diagnostics).

## Results

### TYLCV V2 accumulates in tomato leaves and stems upon whitefly-mediated infection

**Figure 1:**
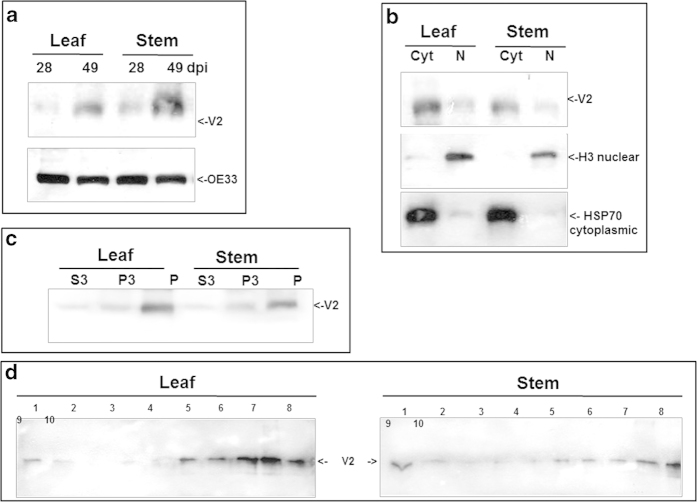
**Detection of TYLCV V2 in tomato leaf and stem 28 and 49 days (dpi) after infection. a**: western blot of V2 and OE33 (chloroplast protein used as an internal marker). **b**: western blot of V2 in leaf and stem tissues at 49 dpi fractioned into cytoplasmic/membrane (Cyt) and nuclear (N) components; cytoplasmic Hsp70 and nuclear histone H3 used as internal markers to assess the fraction purity. **c**: western blot of V2 in leaf and stem tissues of 49 dpi fractionated into insoluble debris and cell wall (P), 3000 *g* pellet (P3) and soluble protein (S3). **d**: western blot analysis of V2, distributed in linear 10-50% sucrose gradients from leaf and stem native protein extracts at 49 dpi; gradients were divided into 10 fractions, 1 (top) to 10 (bottom) and aliquots were subjected to SDS-PAGE.

TYLCV V2 was immuno-detected in protein extracts of tomato leaves and stems using a specific antibody, 28 and 49 days post whitefly-mediated inoculation (dpi) (Fig. 1a). The reacting polypeptide had a 13 kDa molecular mass, in accordance with the protein translated from the V2 ORF. The amount of V2 increased with the progress of infection (28 vs. 49 dpi). The amount of chloroplast protein OE33 used as internal marker did not change significantly upon progress of infection. The proteins were separated into cytoplasmic/membranal and nuclear fractions from leaf and stem; HSP70 and histone H3 were used as markers for these two fractions, respectively. The results showed that V2 was more abundant in cytoplasmic/membranal than in nuclear extracts (shown for 49 dpi, Fig. 1b).

### Most V2 is present in an insoluble form

TYLCV CP was shown previously to be more abundant in insoluble than in soluble protein fractions of tomato leaf and stem^5^,^18^. The extent of solubility of the V2 protein was determined by fractionating infected tomato tissues at 49 dpi in detergent-free buffer into soluble supernatant (S3), insoluble pellet (P3), and cell walls and debris (P) (Fig. 1c). Protein extraction performed earlier than six weeks after the onset of infection yielded too little V2 for this type of analysis. Although V2 was immuno-detected in all fractions, P3 and P contained the highest amounts of the protein (Fig. 1c). These results suggested that most V2 is present in an insoluble state.

Following this observation, we determined the state of V2 aggregation by ultracentrifugation of leaf and stem protein extracts in non-denaturing 10-50% sucrose gradients^28^, allowing protein separation from soluble to aggregates of increasing size, as previously shown for CP of TYLCV and TMV^18^,^28^. Because V2 amounts in protein extracts were low even at the late stages of infection (e.g. 49 dpi), gradient fractions were concentrated 20 times by TCA precipitation prior to western blot immuno-detection. Gradient analyses (Fig. 1d) showed that small amounts of V2 were in a soluble form (fractions 1, 2), while most of the protein was in fractions that contained small (fractions 5, 6), midsize (fractions 7, 8) and large aggregates (fractions 9, 10).

**Figure 2:**
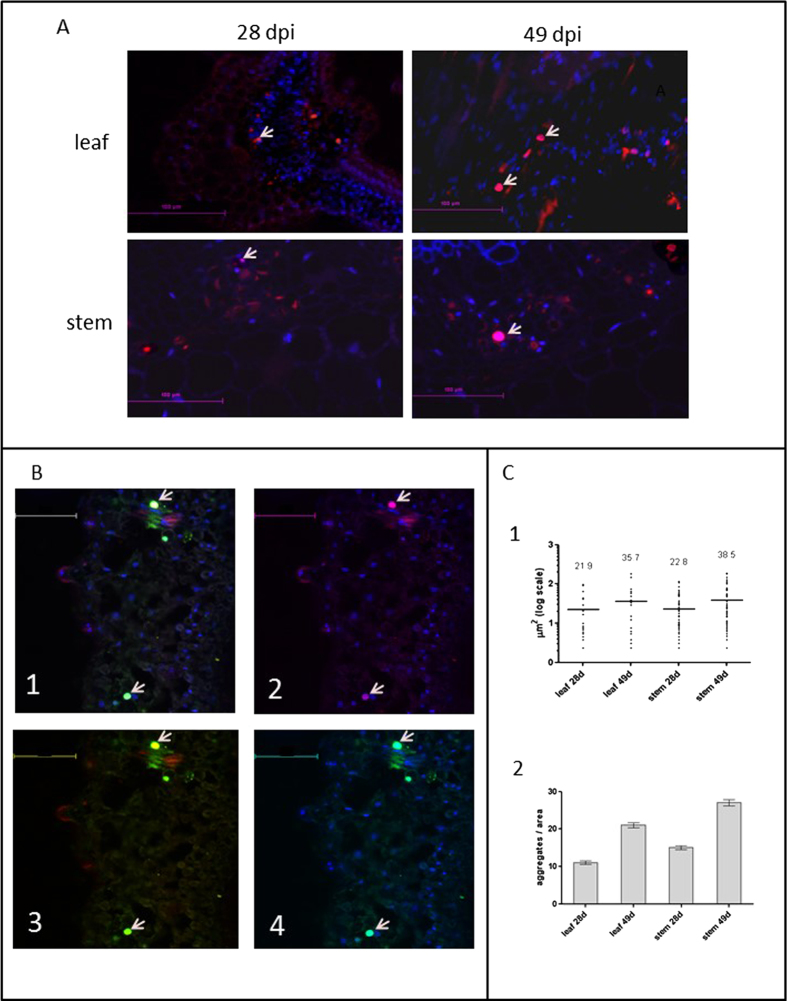
**Immunostaining of V2 in cross sections of midribs of infected tomato leaves and of stems at 28 and 49 dpi. a**: cross sections of midribs of infected tomato leaf and stem; nuclei are DAPI stained (blue); V2 is stained with Cy3-labeled antibody (red); V2 localized in nuclei appears as pink (arrows). Bar: 100 μm. **b**: co-localization of CP and V2 in infected (49 dpi) leaves by triple-stain immunolocalization. 1. Cy-3 labeled V2 (red), Cy5-labeled CP (green) and DAPI-stained nuclei (blue). 2. Cy-3 labeled V2 and DAPI-stained nuclei. 3. Cy-3 labeled V2 and Cy5-labeled CP. 4. Cy5-labeled CP and DAPI-stained nuclei. V2 with CP in nuclei is indicated with arrows. Bar: 100 μm. **c**: cross sections of midribs immunostained with anti-V2 antibody were analyzed with ImageJ. Displayed are the values of counts in 10 leaf areas of 50 mm^2^ and 10 stem areas of 70 mm^2^ in 5 individual experiments for 28 dpi and 48 dpi. 1: size of aggregates. Bars and numbers indicate the mean sizes of aggregates. 2: number of aggregates. Bars: standard error.

These results were confirmed by *in situ* immuno-fluorescent visualization of V2 in leaf and stem sections of symptomatic tomato plants at 28 and 49 dpi. The first appearance of V2 aggregates was detected at 28 dpi. At later stages (49 dpi), the abundance of V2 aggregates increased (Fig. 2a), some of the V2 aggregates were associated with the nucleus (Fig. 2a). Quantification of V2 aggregates (Fig. 2c) indicated that between 28 and 49 dpi, the number of aggregates per area doubled and that their size increased by about 50%. Double *in situ* immuno-detection of V2 and CP revealed that V2 and CP occasionally co-localized in large nuclear inclusions (Fig. 2b, shown for leaf). The low abundance of V2 in the nuclei correlates with the low amounts of V2 in infected tomato tissues.

### V2 binds TYLCV DNA *in vitro* and *in planta*

**Figure 3:**
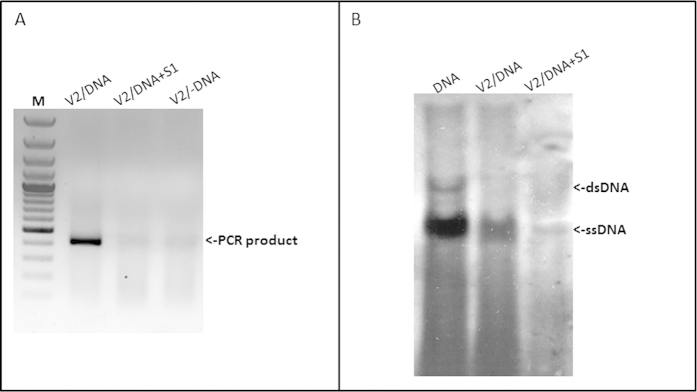
**V2 forms complexes with TYLCV genomic DNA**. Detection of TYLCV DNA-V2 complexes *in vitro* (**a**) and *in planta* (**b**). **a**: PCR detection of viral DNA following immuno-capture with V2 antibody of mixtures of V2 and infected plant DNA; V2/DNA: V2 incubated with untreated DNA, V2/DNA + S1: V2 incubated with DNA treated with S1 nuclease, V2/-DNA: V2 incubated without DNA. M - 100 bp DNA marker. **b**: Southern blot analysis of viral DNA-V2 complexes from infected tomato plants; DNA: DNA from infected plants, V2/DNA: DNA extracted from immunoprecipitated V2 complexes, V2/DNA + S1: DNA extracted from immunoprecipitated V2 complexes and treated with S1 nuclease.

Binding of the V2 protein to TYLCV DNA was demonstrated by IC-PCR, a method previously used to detect TYLCV CP-DNA complexes^29^. In *in vitro* tests, V2 over-expressed in *E. coli* was incubated with DNA extracted from infected plants in PCR tubes coated with anti-V2 antibody. TYLCV DNA bound to V2 was detected by PCR. When the DNA extract was treated with S1 nuclease prior to incubation with V2 (to eliminate the TYLCV genomic ssDNA, but not the dsDNA), the PCR amplicon was not obtained (Fig. 3a). This result suggested that V2 binds TYLCV ssDNA. V2-TYLCV DNA complexes in extracts of infected tomato plants were identified by immuno-precipitation with the anti-V2 antibody followed by Southern blot hybridization (Fig. 3b). When compared with viral DNA (both ss and ds) from infected tomato, it was clear that the viral DNA that immuno-precipitated with V2, was essentially ssDNA; the hybridization signal disappeared after S1 nuclease digestion (Fig. 3b). This result suggested that V2 can make complexes with viral genomic ssDNA *in planta*, but not with its dsDNA replicating form.

### V2:GFP forms aggregates in tomato and in *N. benthamiana* cells

**Figure 4:**
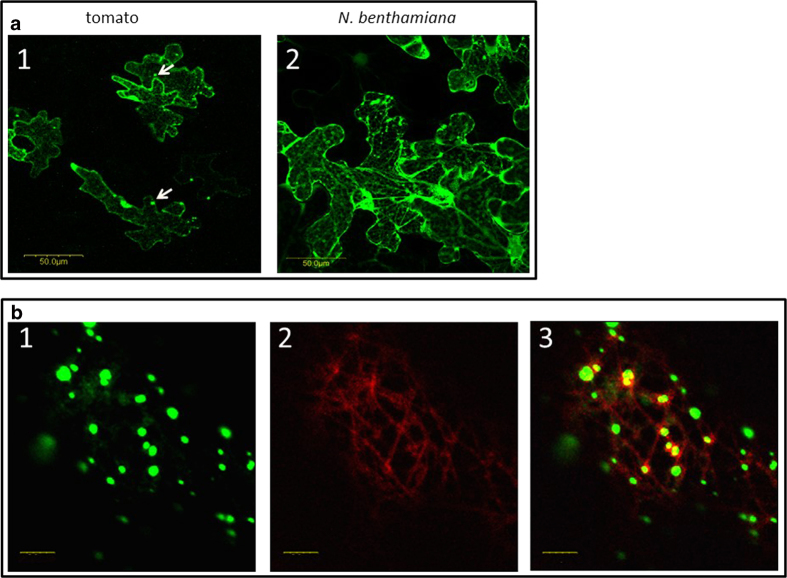
**Expression of fusion V2:GFP in epidermal cells of tomato and *N. benthamiana* leaves**. **a**: tomato (1) and *N. benthamiana* (2) leaves infiltrated with *A. tumefaciens* carrying the V2:GFP expression plasmid. Bar: 50 μm. **b**: *N. benthamiana* leaves infiltrated with A. tumefaciens carrying the V2:GFP and MAP4:RFP expression plasmids: 1. V2:GFP (green); 2. microtubule marker MAP4:RFP (red); 3. merge of V2:GFP and MAP4:RFP, V2 co-localization with microtubules is indicted in yellow. Bar: 5 μm.

A V2:GFP fusion protein was used to determine whether V2 aggregates in the absence of other viral proteins and infection. Following agroinfiltration of the construct, the transiently expressed fluorescent V2:GFP was detected in tomato and in *N. benthamiana* epidermal cells (Fig. 4a). In both plants, V2:GFP appeared as cytoplasmic small aggregates of different sizes throughout the cell, though more cells seemed to express V2:GFP in *N. benthamiana* than in tomato. The V2:GFP fluorescent aggregates resembled the V2 aggregates seen in tomato inoculated with viruliferous whiteflies (Fig. 2), suggesting that V2:GFP expression in *N. benthamiana* is suitable for the analysis of V2 aggregation.

### Disruption of cytoskeleton impairs V2:GFP aggregation

**Figure 5:**
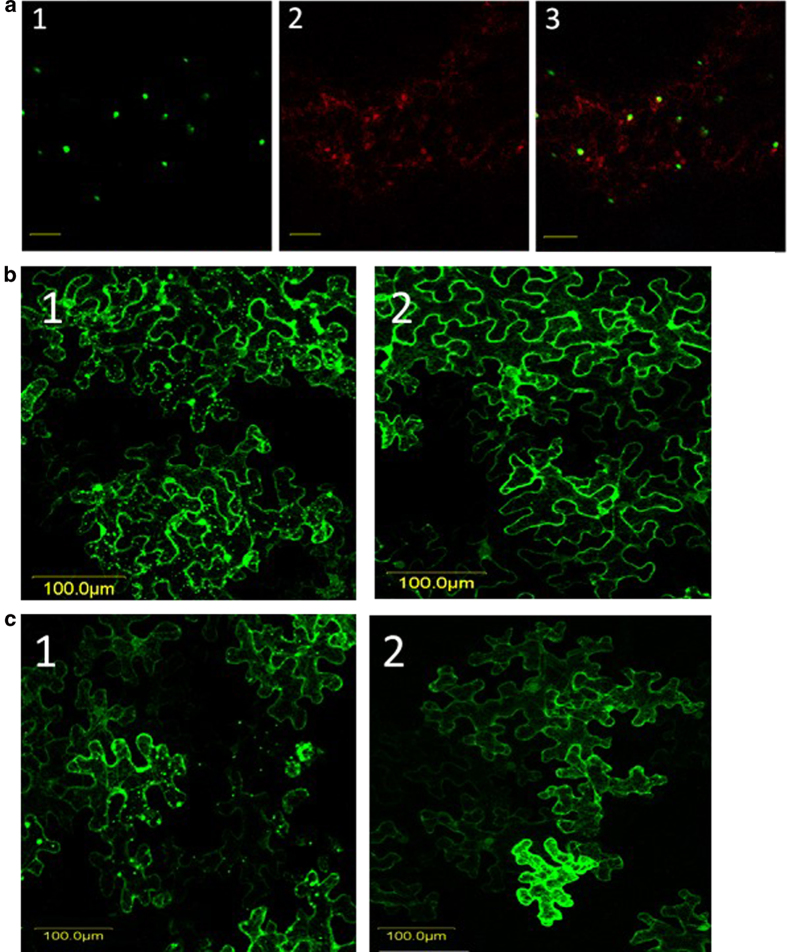
**Disruption of cytoskeleton elements results in changes in V2 aggregation. a**: Inhibition of microtubules with oryzalin results in V2 aggregates of reduced size. Oryzalin treated *N. benthamiana* epidermal cells expressing V2:GFP (green) (1) and the microtubule marker MAP4:RFP (red) (2); 3: merge. Bar: 5 μm. **b**: *N. benthamiana* leaf epidermal cells expressing V2:GFP in untreated (1) and oryzalin treated (2) tissues. Bar: 100 μm. **c**: inhibition of actin filaments prevents aggregation of V2. *N. benthamiana* epidermal cells expressing V2:GFP in untreated (1) and latB treated (2) leaves. Bar: 100 μm.

**Figure 6:**
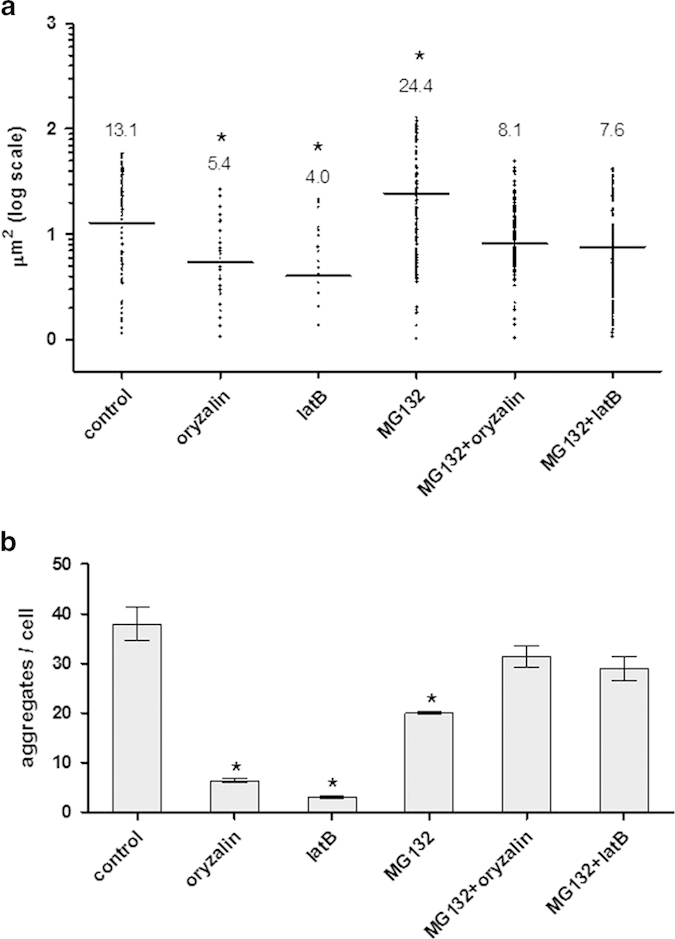
**Inhibition of the cytoskeleton and the 26S proteasome leads to changes in the size and number of V2:GFP aggregates in** ***N. benthamiana*****. a**: sizes of aggregates. Images obtained as described for figs. 5,7 were analyzed with ImageJ. Bars and numbers indicate the mean sizes of aggregates as measured for leaf areas of 50 mm^2^ in 5 independent experiments for each treatment. **b**: average number of aggregates per cell. Displayed are the mean values +/− standard error of counts in leaf areas of 50 mm^2^ in 5 independent experiments for each treatment. Statistical significance between treatments and control were determined by one-way ANOVA (^*^ = P<0.05).

Aggregation of viral proteins often depends on interactions with proteins involved in intracellular movement. Since the cytoskeleton plays an important role in the intracellular movement of viral proteins (reviewed in 3, 10), we tested whether V2:GFP aggregation was dependent on the integrity of microtubules and actin filaments. The potential involvement of microtubules in V2 aggregation was investigated by labeling microtubules with the microtubule marker MAP4:RFP (microtubule binding domain of MAP4 fused to RFP^27^. Co-expression of V2:GFP (green color) and MAP4:RFP (red color) revealed that some V2 aggregates localized in the vicinity of microtubules (yellow color) (Fig. 4b). The involvement of microtubules in V2 aggregation was further studied using oryzalin, a herbicide known to depolymerize microtubules^30^. In *N. benthamiana* leaves infiltrated with V2:GFP and treated with oryzalin, indeed the microtubules were depolymerized as shown by the appearance of the red fluorescence associated with MAP4:RFP (Fig. 5a). While V2:GFP aggregates occurred in the vicinity of microtubules in untreated tissues, in oryzalin-treated leaves V2:GFP appeared as smaller aggregates and fewer co-localized with MAP4:RFP (Fig. 6a, to be compared with Fig. 4b). The observation of a large number of cells showed that in oryzalin-treated cells, the number of aggregates diminished by about 80% and their average size decreased by about 2.5 folds (Figs. 5b,6).

The potential involvement of actin filaments in V2 aggregation was investigated by treating *N. benthamiana* V2:GFP-expressing leaves with latrunculin B (latB), a toxin that inhibits the polymerization of actin microfilaments^31^. While V2:GFP appeared as aggregates of different sizes in untreated leaves, in latB-treated cells the fluorescence associated with the protein was diffuse, and forming a smaller number of aggregates of reduced size (Fig. 5c). Indeed, quantification of a large number of cells showed that in latB-treated cells, the number of aggregates diminished by about 90% and their average size decreased by about 3 folds (Fig. 6). These results indicated that the disruption of microtubule and of actin filaments have a similar effect on V2 aggregation. Therefore, the formation of V2 aggregates of increasing size and their stability depends on the integrity of the actin and microtubule components of the cytoskeleton.

### Inhibition of 26S proteasome results in V2:GFP aggregates of increased size; subsequent inhibition of cytoskeleton elements leads to the re-appearance of small aggregates

**Figure 7:**
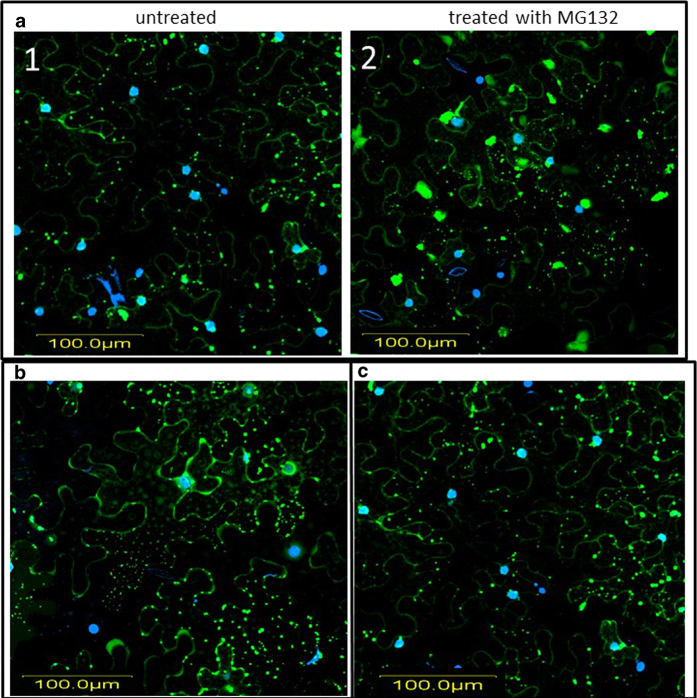
**Inhibition of the 26S proteasome results in the formation of large aggregates; disruption of microtubules or of actin filaments inhibits the delivery of V2 to the proteasome. a**: *N. benthamiana* epidermal cells expressing V2:GFP in untreated (1) and MG132 treated (2) leaf; DAPI stained nuclei appear as blue; V2:GFP appears as green. **b**: and **c**: *N. benthamiana* epidermal cells expressing V2:GFP in MG132 and latB (**b**), and in MG132 and oryzalin (**c**) treated leaves. DAPI stained nuclei appear as blue; V2:GFP appears as green. Bar: 100 μm.

Several plant viral proteins were shown to be degraded by the 26S proteasome^14^,^32^. Since protein aggregation is often accompanied by protein degradation^13^, we addressed the question of whether V2 aggregates were degraded by the 26S proteasome. *N. benthamiana* leaves expressing V2:GFP were treated with the proteasome inhibitor MG132^32^. Compared to untreated cells, the 26S proteosome inhibitor caused a 50% diminution in the number of aggregates, but those remaining exhibited about twice the size of the control V2:GFP aggregates (Figs. 6 and 7a). Since the delivery of proteins to the proteasome often involves the cytoskeleton (reviewed in 13), a potential role of microtubules and actin filaments in V2:GFP degradation by the 26S proteasome was investigated. Leaves expressing V2:GFP were treated with the proteasome inhibitor MG132, together with either oryzalin or latB, to disrupt microtubules and/or actin filaments, respectively. The presence of either cytoskeletal inhibitor interfered with the formation of large V2 aggregates in the absence of proteasome activity; instead, numerous small fluorescent aggregates, which were more similar in size to those present in untreated cells, were formed (Figs. 6,7b and c). These results suggested that the disrupting of either microtubules or actin filaments restrains the delivery of V2 to the 26S proteasome.

### Cytoskeleton and 26S proteasome impairment change the V2 aggregation pattern in TYLCV infected tomato leaves

**Figure 8:**
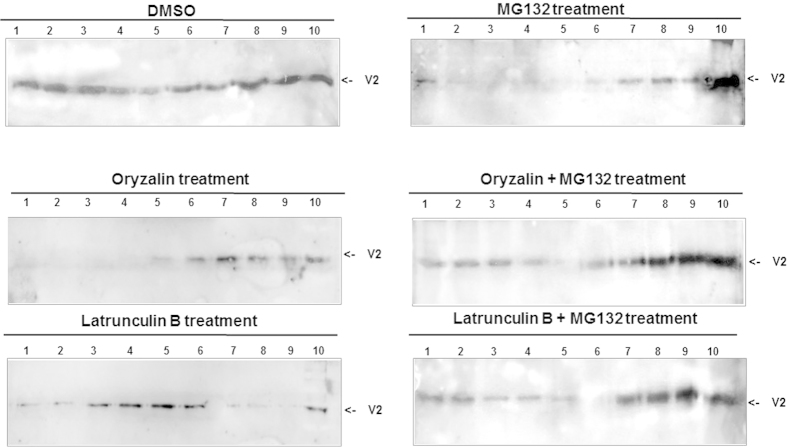
**Inhibition of the cytoskeleton and the 26S proteasome interferes with V2 aggregation in TYLCV-infected tomato.** Western blot analysis of V2, distributed in linear 10-50% sucrose gradients from detached leaves of infected tomato (49 dpi) untreated (DMSO control) or leaves treated with oryzalin, latB, MG132, oryzalin+ MG132 and latB+ MG132. Gradients were divided into 10 fractions, 1 (top) to 10 (bottom) and aliquots were subjected to SDS-PAGE. The experiment was repeated three times with similar results.

To determine whether the proteasome and cytoskeleton inhibitors have similar effects on V2 aggregation during virus infection, leaves of TYLCV-infected tomato plants were treated with oryzalin, latB, and MG132, separately or in combination (oryzalin + MG132 and latB + MG132). Proteins were extracted after 4 days and separated by ultracentrifugation in sucrose gradients, and the viral V2 was immuno-detected on western blots. In samples from tissues with disrupted cytoskeleton, V2 was observed in fractions reflecting aggregates of smaller sizes than in untreated leaves (Fig. 8). Treatment with oryzalin led to a shift of V2 aggregates from mostly mid-sized (fractions 8, 9) to small aggregates (fractions 6, 7). Treatment with latB led to V2 appearance in fractions containing protein complexes (fractions 3-5) and to its disappearance from mid-sized aggregates (fractions 7-9). Following 26S proteasome inhibition by MG132, most of the V2 was associated with large aggregates (fraction 10) (Fig. 8). The combined treatment of 26S proteasome and cytoskeleton inhibitors diminished the effect of MG132, causing the re-appearance of V2 in small/mid-sized aggregates. Such V2 patterns resembled V2 distribution in sucrose gradients of untreated leaf tissue (Fig. 8). These results demonstrated the involvement of the cytoskeleton and the 26S proteasome in the development of V2 aggregation and its turnover in plants naturally infected with TYLCV.

## Discussion

Viral proteins form conspicuous aggregates in the cytoplasm and nucleus of plant cells infected by viruses. Although the role of aggregation was studied extensively in mammalian cells, less is known about the role of aggregation of viral proteins during infection in plant cells. Following the dynamics of aggregation of viral proteins in plant host cells provides new insights into virus-host interactions, especially with respect to the cellular structures involved in the defense against viruses or exploited by viruses (reviewed in 2). In general, aggregates may reflect the presence of local scaffolds for anchoring and accumulation of protein complexes. Such anchored accumulation sites can support virus infection by increasing the local concentration of viral and host components required for replication and assembly, and by shielding the process of replication from host defense. Aggregation of proteins with support of cellular scaffolds may also facilitate innate cellular responses that recognize virus components and target them for storage and degradation^2^. One way to distinguish between these potential roles of aggregates during infection is to identify the cellular structures involved in aggregate formation and to link their function to either viral protein accumulation or degradation.

We have previously reported that the TYLCV CP forms aggregates of increasing size during infection and suggested a functional role for these structures during the development of infection^18^. Here we have studied DNA binding properties and subcellular accumulation pattern of V2, and its dependence on intact cytoskeletal actin and microtubule filaments. V2 seems to be a multifunctional non-structural protein involved the in suppression of gene silencing^23^. Previously it was suggested that V2 is involved in virus intercellular movement in plants^21^, but lately it was shown that TYLCV movement does not require a functional V2^33^ Our analysis of protein extracts indicates that V2 accumulates in leaf and stem cells during viral infection (Figs. 1a and 2), and that it is more abundant in the cytoplasm than in the nucleus (Figs. 1b and 2). Most V2 was detected in insoluble aggregates (Fig. 1c,d). The formation of V2 aggregates was confirmed by immuno-localization of V2 in virus-infected tomato leaves and stems (Fig. 2a,c). To limited extent, we also observed V2 in the nucleus. Those aggregates that occurred in the nucleus also contained CP, and were larger than the cytoplasmic aggregates (Fig. 2b). We have previously postulated that TYLCV nuclear large aggregates, representing VFs, were characteristic of the plant susceptibility to the virus and were an indication of a successful TYLCV invasion^18^. One of the possible functions of V2 in the formation of the nuclear VF might be to enhance nuclear export of viral ssDNA alone or as complexes with CP. Indeed, we show that V2 binds TYLCV ss-, but not ds-DNA *in vitro* and *in vivo* (Fig. 3). These results suggest that V2 is involved in shuttling of TYLCV ssDNA, similarly to the CP.

The association of proteins with elements of the cytoskeleton can be explored using transient expression of fluorescent fusion proteins in a model plant such as *N. benthamiana*. This approach was used for testing the co-localization of TYLCV proteins with markers of the cytoskeleton and discovering the effects of specific cytoskeleton inhibitors. Fluorescence microscopy allowed the detection of V2:GFP fusion protein forming cytoplasmic aggregates (Fig. 4)^21^,^24^,^25^; the same compartment as observed for the majority of untagged V2 protein expressed during infection.

Disruption of cytoskeletal components during transient expression of V2:GFP in *N. benthamiana* as well as during TYLCV infection in tomato prevented the emergence of large/mid-sized V2 aggregates, thus indicating that components of the cytoskeleton are important for V2 aggregation (Figs. 5, 6 and 8). We also demonstrated that the state of V2 aggregates was dependent on the efficiency of 26S proteasome-mediated protein degradation (Figs. 6, 7, 8). Inhibition of the 26S proteasome resulted in the increase in size of V2:GFP aggregates, thus suggesting a role of aggregates as a source of V2 destined for degradation. Importantly, treatment with cytoskeletal inhibitors restored the normal size of the aggregates in the presence of the proteasome inhibitor (Figs. 6, 7, 8) which suggests a role of the intact microtubule and actin cytoskeleton in a pathway by which aggregate formation and protein degradation are linked.

Our observations are consistent with a role of the cytoskeleton in providing localized scaffolds as well as motor-supported transport pathways for the anchorage and accumulation of proteins, thus allowing the localized formation of protein-membrane aggregates with diverse functions^1^,^2^,^3^,^10^. The current results support the notion that the aggregates are linked to proteasome-mediated degradation. A dynamic system of aggregate formation and degradation can be modulated towards more protein accumulation or more protein degradation according to demand. Such a system is convenient for viruses as it allows to accumulate viral and host proteins to form higher order complexes involved in replication (replication factories) and movement. It also allows to control accumulation of viral components (during early infection stages) and undergo enhanced degradation (during later infection stages) through the associated proteasome degradation pathway. This model is supported by various studies in animal systems and plants that imply the role of the cytoskeleton in the formation of aggresomes and the role of aggresomes as centers/VFs for both virus replication and protein accumulation on the one side and protein degradation on the other^13^,^34^. One example is presented by the aggresomal processes observed during TMV infection. Whereas these processes permit the formation of ER-anchored and ER-mobile replication complexes and thus conditions for viral cell-to-cell movement during early infection stages, the same processes allow the maturation of anchored replication sites into VFs for viral replication and virion production, and the subsequent degradation of viral proteins during later stages^35^,^36^. Studies with TMV have shown that the aggresome/VF is linked to proteasome degradation pathway by CDC48, a protein that disaggregates proteins and extracts them from membranes^10^,^11^,^12^. The present study revealed a role of the cytoskeleton and proteasome in the formation and degradation of TYLCV V2 aggregates. The importance of 26S proteasome in degradation of TYLCV CP was recently described^37^. Similar approaches will be applied to find pathways of V2 degradation by cellular machineries. Further studies may reveal additional common steps in the exploitation of cellular cytoskeleton-mediated protein aggregate and turnover mechanisms by viruses as diverse as TMV and TYLCV.

## Author Contributions

A.M. and R.G. designed the experiments. A.M., R.G. and E.B. performed the experiments. A.M., R.G., H.C. A.N. and M.H analyzed the data. A.M., R.G., H.C. A.N. and M.H wrote the manuscript. All authors have read and approved the manuscript.

## Additional Information

**How to cite this article**: Moshe, A. *et al.* The *Tomato yellow leaf curl virus* V2 protein forms aggregates depending on the cytoskeleton integrity and binds viral genomic DNA. *Sci. Rep.* **5**, 9967; doi: 10.1038/srep09967 (2015).
